# The Association of Mixed Mineral Intake Exposure With Gout: A National Cross‐Sectional National Health and Nutrition Examination Survey (NHANES) Study

**DOI:** 10.1002/fsn3.71152

**Published:** 2025-11-09

**Authors:** Rui Lai, Huilin Liu, Shiqi Wang, Xiaofeng Lv, Ying Li

**Affiliations:** ^1^ Chengdu University of Traditional Chinese Medicine School of Acupuncture and Tuina Chengdu China

**Keywords:** bayesian kernel machine regression, calcium, gout, mineral intake, NHANES, quantile g‐computation

## Abstract

Minerals influence urate production, renal clearance, oxidative stress, and systemic inflammation, yet epidemiologic evidence linking mineral intake to gout is limited and rarely considers co‐exposures. We analyzed six NHANES cycles (2007–2018) comprising 22,661 adults ≥ 20 years. After 1:1 propensity‐score matching, usual intakes of nine dietary minerals were divided into quartiles. Survey‐weighted multivariable logistic regression quantified single‐mineral associations with gout. Weighted quantile sum (WQS) regression, quantile g‐computation (qgcomp), and Bayesian kernel machine regression (BKMR) evaluated combined effects and potential interactions. In logistic regression analysis, only calcium showed a consistent inverse, dose‐dependent relation with gout. For the highest versus lowest calcium quartile, the ORs were 0.55 (95% CI 0.37–0.81, *p* = 0.003) in Model 1, 0.52 (95% CI 0.36–0.77, *p* = 0.001) in Model 2, and 0.51 (95% CI 0.36–0.74, *p* < 0.001) in Model 3. No significant associations were observed for phosphorus, magnesium, zinc, iron, copper, potassium, selenium, or sodium (all *p* > 0.05). In mixture analyses, the mineral index was inversely associated with gout: WQS‐OR = 0.88 (95% CI 0.78–0.99) and qgcomp‐OR = 0.87 (95% CI 0.79–0.97). BKMR confirmed an overall inverse exposure‐response function for the combined minerals and identified calcium as the dominant contributor (posterior inclusion probability 0.72). Pair‐wise response surfaces showed no consistent synergistic or antagonistic interactions. Greater calcium intake was associated with reduced gout prevalence in US adults. These findings highlight calcium‐rich foods, particularly low‐fat dairy products, as potential components of gout‐preventive dietary strategies and underscore the utility of mixture modeling for clarifying complex nutritional exposures. Prospective studies are warranted to confirm causality and elucidate underlying mechanisms.

## Graphical abstract

Key findings: Higher dietary calcium intake is associated with a reduced risk of gout in US adults. This inverse relationship was consistent across multiple analytical methods, including logistic regression and mixture models. Calcium emerged as the dominant protective mineral, emphasizing the potential role of calcium‐rich foods, especially dairy, in gout prevention.

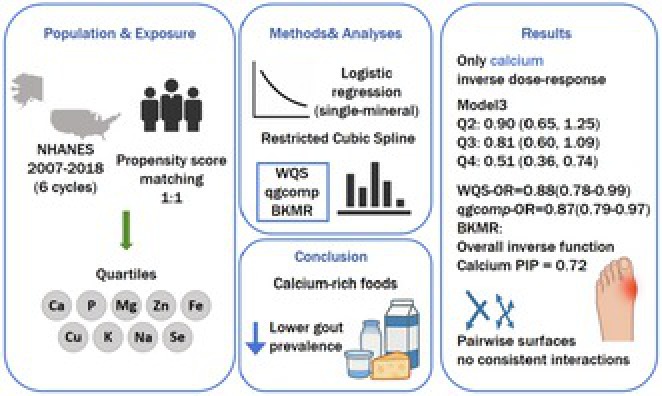

## 1 Introduction

Gout is the leading inflammatory arthritis, affecting millions, and is becoming more common worldwide (DALYs GBD, Collaborators H 2018; Yokose et al. 2023). Persistent high serum urate causes monosodium urate crystals to settle in joints and produce painful flares (McCormick and Choi 2024). Diet strongly influences this process by shaping purine load and uric acid production as well as excretion (Kakutani‐Hatayama et al. 2017). Frequent intake of purine‐rich meat, seafood, alcohol, and sugar‐sweetened drinks increases risk, whereas dairy and many plant foods appear protective (Li, Yu, and Li 2018).

Among dietary factors, minerals deserve particular attention. Calcium, phosphorus, magnesium, iron, zinc, copper, potassium, sodium, and selenium take part in acid–base balance, oxidative defense, renal function, and immune regulation (Adeva and Souto 2011). Epidemiological work has linked higher calcium intake with lower serum urate and fewer gout events, consistent with evidence that dairy promotes renal urate clearance (Zhang et al. 2022). Calcium‐rich diets have been linked to lower serum uric acid and reduced gout incidence in multiple studies (Candido et al. 2022; Dalbeth and Palmano 2011; Zgaga et al. 2012). Results remain mixed, however, largely because most studies have examined each mineral separately and therefore ignored the combined exposures found in real diets (Jiang et al. 2020; Liu et al. 2019; Liu et al. 2022; Pieczynska et al. 2019; Yuan et al. 2022).

We addressed this limitation using data from the National Health and Nutrition Examination Survey collected from 2007 to 2018. Physician‐diagnosed gout was analyzed against usual intakes of the nine minerals in a nationally representative sample of adult participants. Mineral‐specific associations were estimated with multivariable logistic models. To capture joint and possibly nonlinear effects, we also applied mixture approaches that included weighted quantile sum regression (WQS), quantile g‐computation (qgcomp), and Bayesian kernel machine regression (BKMR). This integrated strategy allowed us to test whether higher intakes of individual minerals and particular mineral combinations relate to lower odds of gout in the United States adult population.

## 2 Method

### 2.1 Study Population of NHANES

**FIGURE 1:**
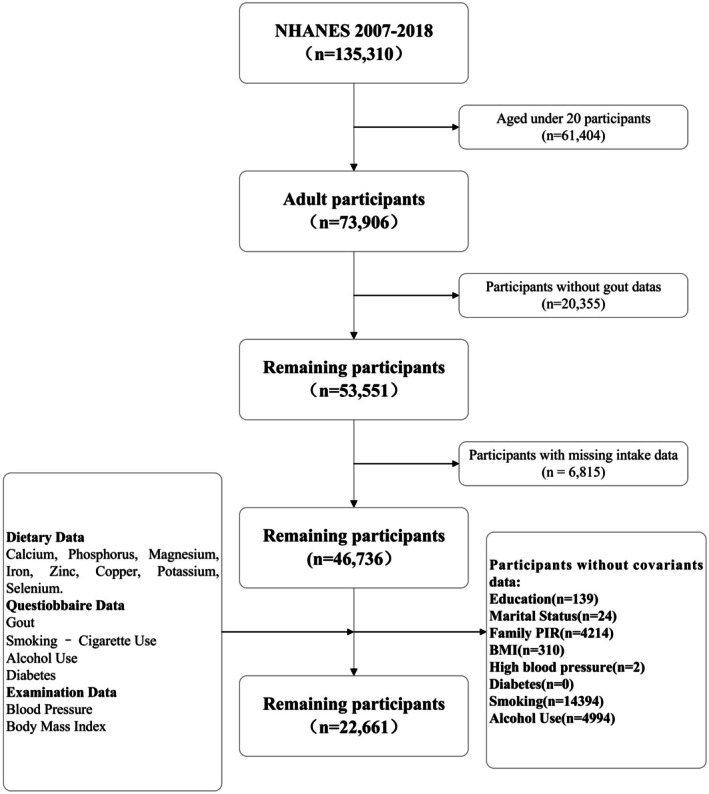
Flowchart.

For this study, we used data from six consecutive NHANES cycles (2007–2018) to construct the cohort. Initially, the cohort consisted of 135,310 US participants. After applying strict exclusion criteria, individuals were removed based on the following: (a) age under 20 years (*n* = 61,404), (b) absence of gout data (*n* = 20,355), (c) missing intake data (*n* = 6815), and (d) missing covariate data, including education level (*n* = 139), marital status (*n* = 24), PIR (*n* = 4214), BMI (*n* = 310), hypertension (*n* = 2), diabetes (*n* = 0), smoking status (*n* = 14,392), and alcohol consumption status (*n* = 4994). After applying these exclusions, the final analysis included 22,661 US participants aged 20 and older. More details in Figure 1.

### 2.2 Assessment of Mineral Intake

Dietary mineral intake data were obtained from two 24 h dietary recall interviews. In the first interview, participants reported all foods and beverages consumed during the previous 24 h while attending the Mobile Examination Center (MEC). A second interview was conducted by telephone 3–10 days later to capture potential day‐to‐day variation. The 24 h dietary recall method provided comprehensive information on participants' food and beverage consumption. Nutrient composition for each reported item was derived from the US Department of Agriculture (USDA) Food and Nutrient Database for Dietary Studies (FNDDS). Based on NHANES protocols, niacin intake was calculated, and the mean values from the two interviews were used to estimate the intake of calcium, phosphorus, magnesium, iron, zinc, copper, potassium, sodium, and selenium.

### 2.3 Assessment of Gout

Gout data were obtained through the Health Status Questionnaire. Specifically, participants were asked, “Has a doctor or other health professional ever told you that you had gout?” Responses of “yes” or “no” were used to categorize participants as having or not having gout, respectively. Previous research has shown that self‐reported gout information is highly accurate.

### 2.4 Covariates

With respect to covariates, we adjusted for several potential confounders, including gender, age, race, marital status, education level, poverty income ratio (PIR), BMI, diabetes, hypertension, smoking, and alcohol consumption.

Hypertension was defined based on four blood pressure measurements. A diagnosis of hypertension was made if any measurement indicated a systolic blood pressure over 140 mmHg or a diastolic blood pressure over 90 mmHg. Diabetes status was determined by three questions: “Has a doctor told you that you have diabetes?” “Are you currently taking insulin?” and “Do you take diabetic pills to lower blood sugar?” Anyone answering “yes” to any of these questions was classified as diabetic. Smoking status is determined by the following questions: “Smoked at least 100 cigarettes in life?” or “Do you now smoke cigarettes?” These responses are categorized as “former,” “now,” or “never.” Alcohol consumption is assessed based on the question “Ever have 4/5 or more drinks every day?”

### 2.5 Statistical Analysis

To control for confounding variables, we applied propensity score matching (PSM). A logistic regression model was employed to estimate the propensity scores, which predicted the probability of each participant being assigned to the gout group. Matching was performed using the nearest neighbor method with a caliper of 0.1, enhancing the matching quality. Balance was evaluated using standardized mean differences (SMD). To account for the NHANES survey design, appropriate statistical weights were applied. In the classification model, mineral intake levels were divided into quartiles, with the lowest quartile (Q1) serving as the reference group. A multivariable logistic regression model was used to examine the relationship between mineral intake and gout. Mineral intake was included in the model as both continuous and quartile variables to explore its potential association with gout. Three models were used to assess the independent relationship between mineral intake and gout: Model 1: Unadjusted; Model 2: Adjusted for demographic characteristics; and Model 3: Adjusted for all the covariates.

Subsequently, the dose–response relationship between multi‐mineral intake and gout risk was analyzed using RCS regression. To account for the joint effects of multiple minerals, we employed WQS regression. We fitted the model using quartile independent variables and set the ratio of training and validation sets to 6:4, performing 10,000 bootstrap iterations. The overall effect direction was predefined as negative. Mineral intake with an estimated weight > 0.111 (1/9) was considered to have a significant contribution to the joint effect. Next, we applied qgcomp analysis to evaluate the combined effects of mineral exposures while adjusting for potential confounders, using the R package qgcomp. The model was fitted with quartile‐transformed exposures, and component weights were estimated for both positive and negative directions. Finally, BKMR was employed to model the complex, non‐linear, and interactive relationships between mineral intake and gout risk. A Gaussian kernel function was used with 50,000 Markov Chain Monte Carlo (MCMC) iterations to ensure model convergence and stability. Continuous variables were expressed as the mean ± standard deviation (SD), while categorical variables were reported as percentages. Statistical analyses were conducted using R (4.4.2), with a significance threshold set at a *p* value of less than 0.05.

## 3 Results

### 3.1 Baseline Profile Before Propensity‐Score Matching

The raw NHANES sample comprised 22,661 adults, of whom 1136 (5.01%) self‐reported physician‐diagnosed gout. Compared with the 21,525 gout‐free participants, the gout group was appreciably older (≥ 60 years: 66.0% vs. 30.8%), more often male (75.2% vs. 51.3%), and displayed a higher prevalence of cardiometabolic comorbidities such as diabetes (33.7% vs. 12.5%) and hypertension (81.7% vs. 43.9%). Marked imbalances were also observed across most of the covariates (*p* < 0.001), confirming substantial selection bias between the comparison groups (Table S1).

### 3.2 Matched Cohort Construction and Balance Assessment

Following 1:1 PSM, 1136 gout cases were matched to 1136 non‐gout controls. Post‐matching comparisons revealed a minor imbalance in race (*p* = 0.018), while all other covariates were well balanced (all *p* > 0.05). Importantly, the absolute standardized mean differences for all matched variables were below the conventional 10% threshold, indicating that the matching procedure effectively reduced confounding and yielded a cohort appropriate for subsequent causal analyses (Figure S1, Table S2).

### 3.3 The Association Between Mineral Intakes and Gout

Logistic regression analyses performed on quartiles of intake showed a significant association between calcium intake and gout risk only (Table 1). In all models, the highest quartile of calcium intake was significantly negatively associated with gout risk (Model 1: OR = 0.55, 95% CI: 0.37–0.81, *p* = 0.003; Model 2: OR = 0.52, 95% CI: 0.36–0.77, *p* = 0.001; Model 3: OR = 0.51, 95% CI: 0.36–0.74, *p* < 0.001). The higher the calcium intake, the lower the risk of gout. In contrast, phosphorus, magnesium, iron, copper, zinc, potassium, sodium, and selenium intakes were not significantly associated with gout risk (*p* > 0.05). The corresponding Restricted Cubic Spline (RCS) curves are shown in Figure S2.

**TABLE 1:** Results of multivariate logistic regression analyses. Q1 (lowest quartile) was used as the reference category in all models.

Characteristics	Model 1	P1	Model 2	P2	Model 3	P3
Calcium						
Q1	Ref	Ref	Ref	Ref	Ref	Ref
Q2	0.93 (0.67, 1.28)	0.644	0.91 (0.66, 1.26)	0.569	0.90 (0.65, 1.25)	0.523
Q3	0.86 (0.63, 1.17)	0.334	0.83 (0.61, 1.13)	0.238	0.81 (0.60, 1.09)	0.157
Q4	0.55 (0.37, 0.81)	0.003	0.52 (0.36, 0.77)	0.001	0.51 (0.36, 0.74)	< 0.001
*p* for trend		0.003		< 0.001		< 0.001
Phosphorus						
Q1	Ref	Ref	Ref	Ref	Ref	Ref
Q2	0.87 (0.60, 1.25)	0.448	0.86 (0.59, 1.24)	0.406	0.85 (0.59, 1.23)	0.388
Q3	0.78 (0.52, 1.17)	0.225	0.76 (0.50, 1.16)	0.198	0.75 (0.49, 1.14)	0.179
Q4	0.70 (0.46, 1.05)	0.082	0.68 (0.45, 1.01)	0.058	0.67 (0.45, 1.00)	0.052
*p* for trend		0.068		0.046		0.041
Magnesium						
Q1	Ref	Ref	Ref	Ref	Ref	Ref
Q2	0.92 (0.61, 1.40)	0.694	0.93 (0.61, 1.43)	0.745	0.94 (0.61, 1.45)	0.787
Q3	0.92 (0.65, 1.31)	0.651	0.92 (0.64, 1.32)	0.651	0.95 (0.66, 1.38)	0.788
Q4	0.69 (0.44, 1.07)	0.098	0.67 (0.43, 1.05)	0.078	0.67 (0.43, 1.06)	0.088
*p* for trend		0.073		0.056		0.067
Iron						
Q1	Ref	Ref	Ref	Ref	Ref	Ref
Q2	1.17 (0.80, 1.71)	0.403	1.16 (0.79, 1.70)	0.441	1.17 (0.79, 1.71)	0.429
Q3	0.82 (0.56, 1.20)	0.303	0.82 (0.56, 1.21)	0.315	0.82 (0.56, 1.20)	0.301
Q4	0.75 (0.49, 1.14)	0.175	0.75 (0.49, 1.15)	0.183	0.75 (0.48, 1.16)	0.191
*p* for trend		0.065		0.070		0.074
Zinc						
Q1	Ref	Ref	Ref	Ref	Ref	Ref
Q2	0.91 (0.62, 1.33)	0.617	0.89 (0.61, 1.29)	0.528	0.89 (0.61, 1.29)	0.521
Q3	0.86 (0.59, 1.24)	0.403	0.84 (0.57, 1.22)	0.346	0.82 (0.56, 1.19)	0.285
Q4	0.67 (0.44, 1.04)	0.072	0.66 (0.43, 1.03)	0.068	0.66 (0.42, 1.02)	0.063
*p* for trend		0.069		0.067		0.059
Copper						
Q1	Ref	Ref	Ref	Ref	Ref	Ref
Q2	0.92 (0.61, 1.40)	0.708	0.92 (0.61, 1.40)	0.694	0.92 (0.60, 1.39)	0.684
Q3	0.83 (0.54, 1.26)	0.378	0.84 (0.54, 1.29)	0.413	0.84 (0.55, 1.29)	0.420
Q4	0.96 (0.58, 1.58)	0.866	0.96 (0.58, 1.59)	0.871	0.95 (0.58, 1.57)	0.847
*p* for trend		0.834		0.865		0.843
Sodium						
Q1	Ref	Ref	Ref	Ref	Ref	Ref
Q2	0.96 (0.64, 1.43)	0.825	0.96 (0.64, 1.44)	0.839	0.96 (0.64, 1.44)	0.837
Q3	0.80 (0.56, 1.13)	0.208	0.80 (0.57, 1.14)	0.217	0.79 (0.56, 1.12)	0.182
Q4	0.89 (0.57, 1.40)	0.619	0.89 (0.56, 1.42)	0.630	0.89 (0.57, 1.39)	0.604
*p* for trend		0.537		0.544		0.511
Potassium						
Q1	Ref	Ref	Ref	Ref	Ref	Ref
Q2	0.87 (0.59, 1.28)	0.489	0.88 (0.59, 1.30)	0.510	0.89 (0.60, 1.32)	0.553
Q3	0.79 (0.54, 1.15)	0.215	0.79 (0.53, 1.19)	0.254	0.80 (0.54, 1.21)	0.288
Q4	0.74 (0.49, 1.12)	0.151	0.73 (0.47, 1.12)	0.145	0.74 (0.48, 1.14)	0.169
*p* for trend		0.124		0.121		0.143
Selenium						
Q1	Ref	Ref	Ref	Ref	Ref	Ref
Q2	0.84 (0.56, 1.26)	0.403	0.85 (0.57, 1.26)	0.412	0.85 (0.57, 1.27)	0.419
Q3	1.12 (0.76, 1.65)	0.550	1.16 (0.79, 1.71)	0.447	1.16 (0.79, 1.72)	0.440
Q4	1.00 (0.64, 1.58)	0.992	1.03 (0.65, 1.63)	0.896	1.04 (0.66, 1.64)	0.861
*p* for trend		0.717		0.615		0.574

**TABLE 2:** Combined effect of nine mineral intakes on gout derived from WQS and qgcomp modeling.

Model	OR (95% CI)	*p*
WQS model	0.88 (0.78–0.99)	0.031
qgcomp model	0.87 (0.79–0.97)	0.009

### 3.4 WQS and qgcomp Regression Analyses

When considered jointly, higher intakes of the nine minerals were linked to a lower likelihood of gout. In the WQS regression, every one‐unit increase in the WQS index corresponded to a 12% reduction in the odds of gout (OR = 0.88, 95% CI: 0.78–0.99; *p* = 0.031) (Table 2). The qgcomp algorithm produced an almost identical estimate (OR = 0.87, 95% CI: 0.79–0.97; *p* = 0.009) after the same covariate adjustment set, reinforcing the robustness of the inverse association (Table 2).

**FIGURE 2:**
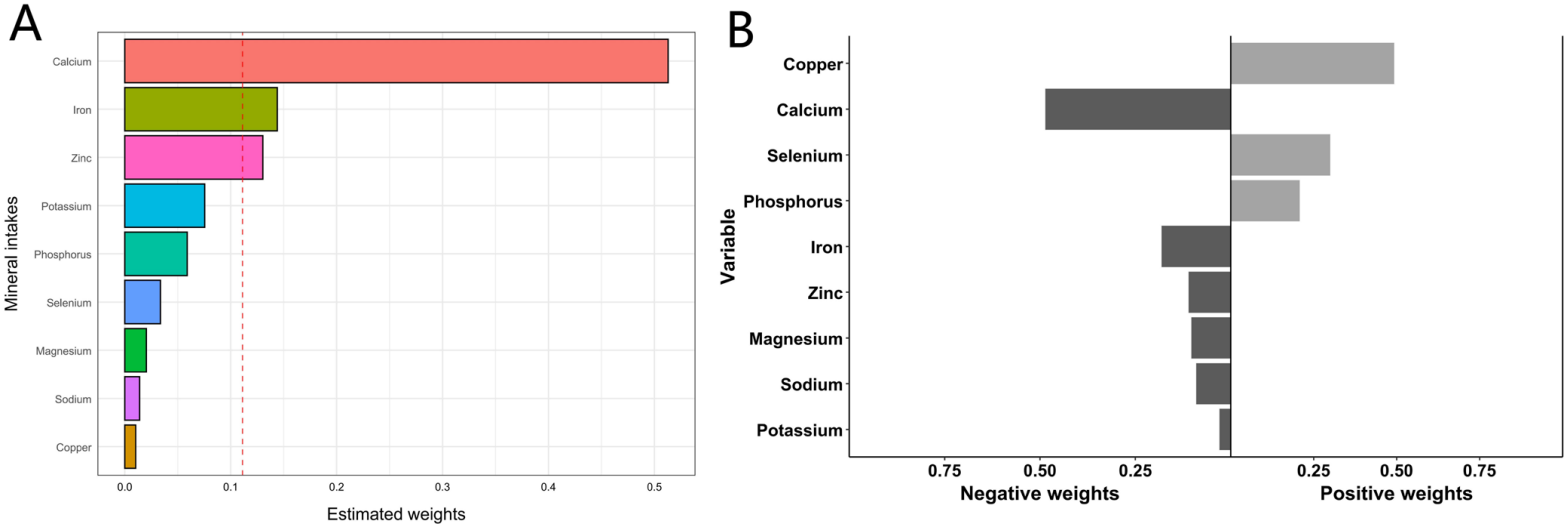
(A). The WQS model weights and (B) the qgcomp model weights.

In the WQS model (Figure 2A), the weights of the nine minerals are calcium (0.513), iron (0.144), zinc (0.130), potassium (0.075), phosphorus (0.059), selenium (0.034), magnesium (0.020), sodium (0.014), and copper (0.010). The qgcomp weighting curve echoes this hierarchy: calcium again dominates the negative (protective) direction, followed by a cluster of trace elements led by iron and zinc (Figure 2B).

### 3.5 BKMR Regression Analyses

**FIGURE 3:**
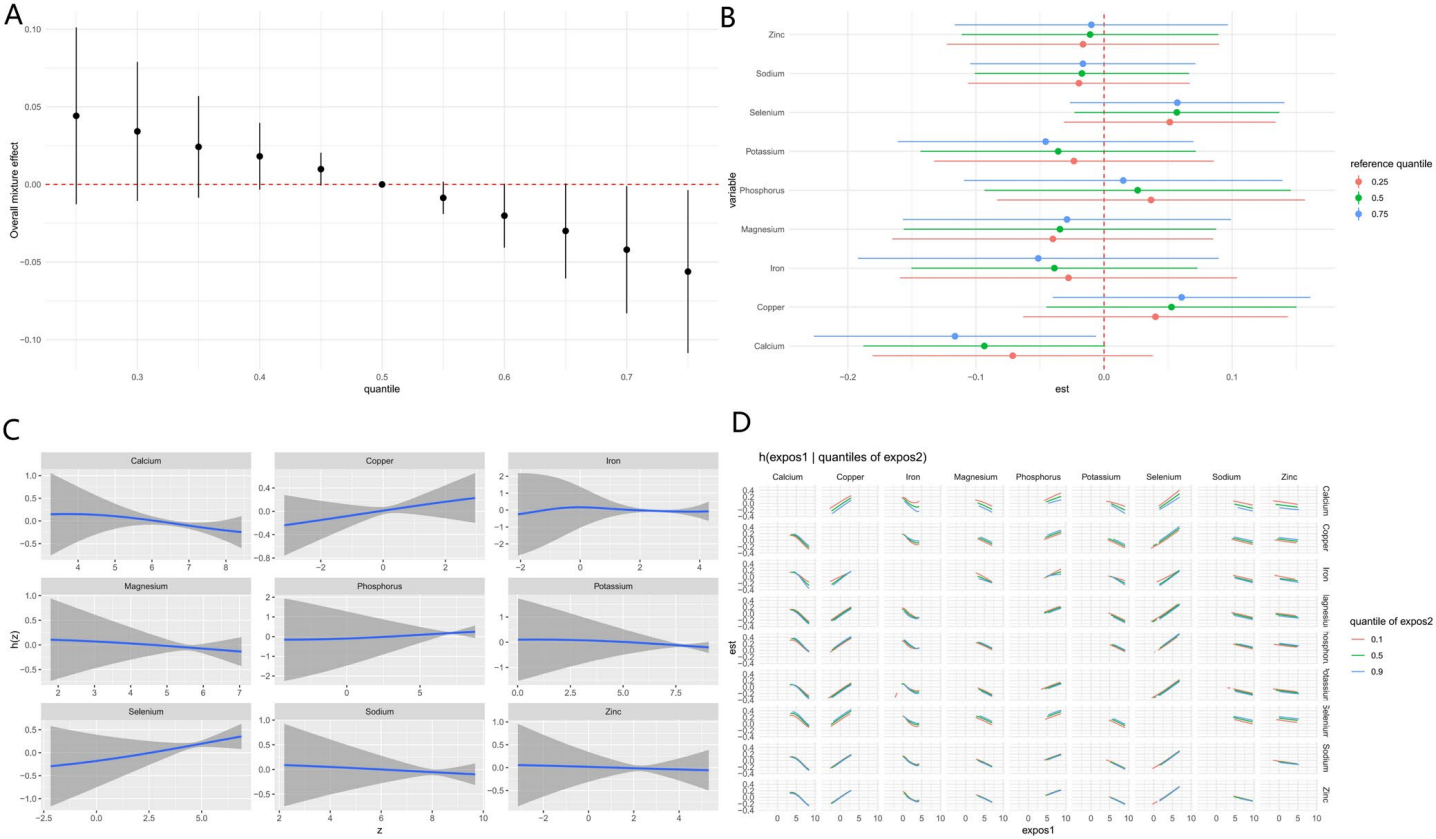
(A) Overall association of mineral mixtures with gout in the BKMR model. (B) Associations of single mineral intakes with gout risk were estimated by BKMR models in the total population and subgroups, when all other mineral intakes were held at their corresponding 25th (red), 50th (green), or 75th (blue) percentiles, respectively. (C) Univariate exposure–response function curves for the association of single mineral intake exposure with gout by the BKMR model. (D) Bivariate exposure–response functions for each mineral intake were assessed with BKMR models when exposure to mineral intake was at different quantiles (10th, 50th, and 90th), and other mineral intakes were fixed at their median levels. Models were adjusted for gender, age, race, education, income, marital status, drinking, smoking, BMI, diabetes, and hypertension.

As shown in Figure 3A, a higher combined intake of the nine minerals corresponded to a modest reduction in gout risk. We further analyzed the relationship between single exposure and gout and found that a statistically significant negative correlation between calcium intake and gout risk was consistently observed (Figure 3B). Posterior inclusion probabilities (PIPs) reinforced this pattern by pinpointing calcium as the principal driver of the association (PIP = 0.72), with iron a distant second (PIP ≈0.40) and all other minerals contributing marginally (PIPs < 0.30) (Table S3). As illustrated by the univariate exposure–response functions in Figure 3C. Finally, bivariate exposure–response surfaces (Figure 3D) showed no consistent synergistic or antagonistic interactions between any mineral pairs, suggesting that the observed benefit stems chiefly from the additive effect of individual minerals, especially calcium, rather than from pairwise combinations.

## 4 Discussion

In this nationally representative cross‐sectional study, higher dietary calcium intake was consistently associated with a lower likelihood of gout. Across multiple analytic approaches, including multivariable logistic regression, WQS, qgcomp, and BKMR, calcium emerged as the most consistently protective mineral among the nine examined. This robust inverse association supports earlier findings linking higher dairy intake, a major source of calcium, to reduced gout risk (Choi et al. 2004; Dalbeth and Palmano 2011). Importantly, our study builds on previous work by isolating the independent contribution of calcium in the context of overall mineral intake. The protective relationship remained significant even after adjusting for a wide range of confounders and accounting for the joint effects of other minerals.

Several biological mechanisms may explain the observed protective effect of calcium against gout. Calcium‐rich foods, particularly low‐fat dairy products, have been shown to promote uric acid excretion and lower serum urate concentrations. Clinical trial evidence supports this: one randomized study found that supplementing approximately 770 mg/day of calcium (via skim milk or calcium citrate) reduced serum urate levels by about 15%, suggesting a direct urate‐lowering effect of calcium itself (Candido et al. 2022). One plausible mechanism is enhanced renal clearance of urate. Studies have demonstrated that calcium intake influences serum urate levels. For instance, a short‐term study in patients with idiopathic urolithiasis reported increased uric acid excretion following intravenous calcium chloride administration (Dunzendorfer and Schmidt‐Gayk 1981). Similarly, calcium‐based phosphate binders used in patients with end‐stage renal disease led to modest reductions in serum urate concentrations (Garg et al. 2005). Calcium may also exert effects in the gastrointestinal tract. It acts as a chelator of oxalates, which are components of urate crystals (Lonsdale 1968; Lonsdale and Mason 1966). Low calcium intake increases oxalate absorption, potentially raising the risk of calcium oxalate crystal formation (Ma et al. 2024). Supporting this, a randomized controlled trial found that higher calcium intake was associated with significantly lower levels of both serum urate and ionic calcium compared to lower intake groups (Candido et al. 2022). Additionally, dietary patterns that emphasize calcium, such as the DASH diet, which is also rich in potassium and magnesium, have been linked to lower gout risk. This further reinforces the potential role of calcium as part of a broader dietary strategy to reduce hyperuricemia and gout risk (Rai et al. 2017).

Calcium may also play a role in modulating systemic acid–base balance. By helping to neutralize diet‐induced metabolic acidosis, higher calcium intake can contribute to a more alkaline internal environment, which enhances uric acid solubility and promotes its excretion (Curhan et al. 1993; Curhan et al. 1997). In contrast, acidic conditions favor urate crystal formation and deposition (Adeva and Souto 2011). Thus, the alkalinizing effect of calcium‐rich diets may help reduce a key physiological trigger for gout. Although genetic evidence on calcium's direct role in gout remains inconclusive, the consistency of our findings with clinical and dietary studies supports a genuine protective association (Chen et al. 2021). This suggests that dietary calcium, rather than systemic calcium regulation, is the more relevant factor. It may serve as an indicator of dairy‐rich, urate‐lowering dietary patterns or exert its effects through interactions with other nutrients commonly found in calcium‐rich foods.

Although multivariate logistic regression of other mineral intakes did not show statistically significant results in our study, related studies suggest that they may influence the onset and progression of gout. For example, magnesium has been associated with a lower prevalence of hyperuricemia in previous studies, and magnesium deficiency may impair uric acid metabolism (Cao et al. 2024; Zhang and Qiu 2018). Potassium, found abundantly in fruits and vegetables, may contribute to the protective effects of plant‐rich diets by reducing net acid load and enhancing renal urate excretion (Chan et al. 2024; Li, Guo, et al. 2018). Trace minerals such as zinc, copper, and selenium play complex roles in oxidative stress and inflammation, both of which are implicated in gout pathophysiology. Zinc, a cofactor for key antioxidant enzymes, has shown urate‐lowering potential in certain settings (Marreiro et al. 2017; Zhang et al. 2018). Epidemiological and experimental data suggest that adequate zinc intake may help regulate serum uric acid levels, possibly by attenuating oxidative mechanisms that drive urate overproduction (Powell 2000; Umeki et al. 1986; Vijayaraghavan et al. 2012). In our study, higher zinc intake was associated with a modest, though statistically nonsignificant, reduction in gout risk, consistent with a potential protective role. Conversely, copper has been positively associated with hyperuricemia in some prior studies. Elevated serum copper levels may reflect increased oxidative stress or inflammation, both of which can promote urate accumulation (Jiang et al. 2020). Selenium, another antioxidant trace element, remains poorly studied in the context of gout. While some reports link higher selenium exposure to increased hyperuricemia risk (Pieczynska et al. 2019), our findings showed no significant association between selenium or copper intake and gout. Sodium intake also showed no meaningful relationship with gout risk in our analysis. Although high sodium consumption can influence blood pressure and kidney function, both of which are related to uric acid regulation, the direct effects of sodium on urate handling appear limited or confounded by other dietary factors (Graudal et al. 2020). Taken together, these findings underscore calcium as the most consistently protective mineral in relation to gout, while the roles of other minerals appear more nuanced or negligible after adjusting for dietary and lifestyle variables.

Several limitations should be considered when interpreting our findings. First, dietary intake was assessed by 24 h recall, which is subject to measurement error and day‐to‐day variability. Single‐day intake may not perfectly capture habitual mineral consumption, potentially leading to misclassification. However, any non‐differential misclassification would likely bias results toward the null, meaning the true associations might be even stronger. Second, gout status in NHANES was based on self‐report of a physician diagnosis. While self‐reported gout is generally considered reliable, there is a possibility of misreporting or undiagnosed cases; nonetheless, such misclassification is also likely non‐differential. Lastly, the generalizability of our findings may be limited to populations with similar dietary behaviors and gout risk profiles as contemporary US adults. Different ethnic diets or lower‐calcium populations might exhibit different associations, and thus, cross‐cultural validation is warranted.

Several limitations should be considered when interpreting our findings. First, dietary intake was assessed by 24 h recall, which is subject to measurement error and day‐to‐day variability. Single‐day intake may not perfectly capture habitual mineral consumption, potentially leading to misclassification. However, any non‐differential misclassification would likely bias results toward the null, meaning the true associations might be even stronger. Second, gout status in NHANES was based on self‐report of a physician diagnosis. While self‐reported gout is generally considered reliable, there is a possibility of misreporting or undiagnosed cases; nonetheless, such misclassification is also likely non‐differential. Third, because the present study is cross‐sectional in design, the temporal relationship between dietary calcium intake and gout cannot be established. Therefore, causal inferences—such as suggesting that higher calcium intake prevents gout—should not be drawn from these findings. Lastly, the generalizability of our findings may be limited to populations with similar dietary behaviors and gout risk profiles as contemporary US adults. Different ethnic diets or lower‐calcium populations might exhibit different associations, and thus, cross‐cultural validation is warranted.

## 5 Conclusion

In conclusion, this study provides robust evidence that higher dietary calcium intake is associated with a lower risk of gout in a nationally representative US population, even after accounting for the intake of other essential minerals. This inverse association was consistent across multiple analytical approaches and aligns with existing evidence linking dietary patterns, particularly those rich in dairy, with reduced gout risk. While the associations for other minerals were weaker and less consistent, our findings support the value of a mineral‐rich, balanced diet, with particular emphasis on adequate calcium intake from dairy or comparable sources, as a potentially beneficial strategy for gout prevention and management. Future prospective studies and randomized controlled trials are warranted to clarify the causal role of calcium and to explore how the overall mineral composition of the diet influences gout risk.

## Author Contributions

R.L. wrote the main manuscript section and performed conceptualization, methodology, and formal analysis; H.L. and S.W. performed formal analysis, data curation, and visualization; X.L. performed data curation and visualization; Y.L. performed supervision, writing review, and editing. All authors contributed to the article and approved the final version of the manuscript.

## Ethics Statement

This study was approved by the Research Ethics Review Board of the US National Center for Health Statistics, and all participants provided informed consent to participate in the NHANES study.

## Consent

All authors have approved the submission and confirm that the work is original, has not been published, and is not under consideration elsewhere.

## Conflicts of Interest

The authors declare no conflicts of interest.

## Supporting information


**Table S1:**Baseline characteristics of the study sample.
**Table S2:** The weighted basic characteristics of the study sample after PSM.
**Table S3:** Posterior inclusion probabilities (PIPs) estimates for all mineral intake exposures.
**Figure S1:** Plot of standardized mean differences before and after propensity score matching for baseline covariates.
**Figure S2:** Restricted cubic spline (RCS) plots of multimineral intake on gout.

## Data Availability

The datasets used in this study are freely available for download from sources in the public domain: NHANES (https://www.cdc.gov/nchs/nhanes/index.htm).

## References

[fsn371152-bib-0001] Adeva, M. M. , and G. Souto . 2011. “Diet‐Induced Metabolic Acidosis.” Clinical Nutrition 30: 416–421. 21481501 10.1016/j.clnu.2011.03.008

[fsn371152-bib-0002] Candido, F. G. , R. D. M. Alves , D. M. O. Freitas , et al. 2022. “Urate‐Lowering Effect of Calcium Supplementation: Analyses of a Randomized Controlled Trial.” Clinical Nutrition ESPEN 49: 86–91. 35623880 10.1016/j.clnesp.2022.02.121

[fsn371152-bib-0003] Cao, X. , H. Feng , and H. Wang . 2024. “Magnesium Depletion Score and Gout: Insights From NHANES Data.” Frontiers in Nutrition 11: 1485578. 39639938 10.3389/fnut.2024.1485578 PMC11617175

[fsn371152-bib-0004] Chan, R. J. , N. Parikh , S. Ahmed , M. Ruzicka , and S. Hiremath . 2024. “Blood Pressure Control Should Focus on More Potassium: Controversies in Hypertension.” Hypertension 81: 501–509. 37641923 10.1161/HYPERTENSIONAHA.123.20545

[fsn371152-bib-0005] Chen, Y. , V. Forgetta , J. B. Richards , and S. Zhou . 2021. “Health Effects of Calcium: Evidence From Mendelian Randomization Studies.” JBMR Plus 5: e10542. 34761146 10.1002/jbm4.10542 PMC8567492

[fsn371152-bib-0006] Choi, H. K. , K. Atkinson , E. W. Karlson , W. Willett , and G. Curhan . 2004. “Purine‐Rich Foods, Dairy and Protein Intake, and the Risk of Gout in Men.” New England Journal of Medicine 350: 1093–1103. 15014182 10.1056/NEJMoa035700

[fsn371152-bib-0007] Curhan, G. C. , W. C. Willett , E. B. Rimm , and M. J. Stampfer . 1993. “A Prospective Study of Dietary Calcium and Other Nutrients and the Risk of Symptomatic Kidney Stones.” New England Journal of Medicine 328: 833–838. 8441427 10.1056/NEJM199303253281203

[fsn371152-bib-0008] Curhan, G. C. , W. C. Willett , F. E. Speizer , D. Spiegelman , and M. J. Stampfer . 1997. “Comparison of Dietary Calcium With Supplemental Calcium and Other Nutrients as Factors Affecting the Risk for Kidney Stones in Women.” Annals of Internal Medicine 126: 497–504. 9092314 10.7326/0003-4819-126-7-199704010-00001

[fsn371152-bib-0009] Dalbeth, N. , and K. Palmano . 2011. “Effects of Dairy Intake on Hyperuricemia and Gout.” Current Rheumatology Reports 13: 132–137. 21188562 10.1007/s11926-010-0160-8

[fsn371152-bib-0010] DALYs GBD, Collaborators H . 2018. “Global, Regional, and National Disability‐Adjusted Life‐Years (DALYs) for 359 Diseases and Injuries and Healthy Life Expectancy (HALE) for 195 Countries and Territories, 1990‐2017: A Systematic Analysis for the Global Burden of Disease Study 2017.” Lancet 392: 1859–1922. 30415748 10.1016/S0140-6736(18)32335-3 PMC6252083

[fsn371152-bib-0011] Dunzendorfer, U. , and H. Schmidt‐Gayk . 1981. “Parathyroid Hormone, cAMP, Electrolytes and Uric Acid After High Dose CaCl2 in Patients With Idiopathic Stone Formation.” Endokrinologie 77: 353–359. 6268394

[fsn371152-bib-0012] Garg, J. P. , S. Chasan‐Taber , A. Blair , et al. 2005. “Effects of Sevelamer and Calcium‐Based Phosphate Binders on Uric Acid Concentrations in Patients Undergoing Hemodialysis: A Randomized Clinical Trial.” Arthritis and Rheumatism 52: 290–295. 15641045 10.1002/art.20781

[fsn371152-bib-0013] Graudal, N. A. , T. Hubeck‐Graudal , and G. Jurgens . 2020. “Effects of Low Sodium Diet Versus High Sodium Diet on Blood Pressure, Renin, Aldosterone, Catecholamines, Cholesterol, and Triglyceride.” Cochrane Database of Systematic Reviews 12: CD004022. 33314019 10.1002/14651858.CD004022.pub5 PMC8094404

[fsn371152-bib-0014] Jiang, T. , D. Xie , J. Wu , et al. 2020. “Association Between Serum Copper Levels and Prevalence of Hyperuricemia: A Cross‐Sectional Study.” Scientific Reports 10: 8687. 32457333 10.1038/s41598-020-65639-0 PMC7250918

[fsn371152-bib-0015] Kakutani‐Hatayama, M. , M. Kadoya , H. Okazaki , et al. 2017. “Nonpharmacological Management of Gout and Hyperuricemia: Hints for Better Lifestyle.” American Journal of Lifestyle Medicine 11: 321–329. 30202351 10.1177/1559827615601973 PMC6125106

[fsn371152-bib-0016] Li, F. , H. Guo , J. Zou , et al. 2018. “The Association of Urinary Sodium and Potassium With Renal Uric Acid Excretion in Patients With Chronic Kidney Disease.” Kidney and Blood Pressure Research 43: 1310–1321. 30099444 10.1159/000492590

[fsn371152-bib-0017] Li, R. , K. Yu , and C. Li . 2018. “Dietary Factors and Risk of Gout and Hyperuricemia: A Meta‐Analysis and Systematic Review.” Asia Pacific Journal of Clinical Nutrition 27: 1344–1356. 30485934 10.6133/apjcn.201811_27(6).0022

[fsn371152-bib-0018] Liu, Y. , J. Zhu , L. Xu , B. Wang , W. Lin , and Y. Luo . 2022. “Copper Regulation of Immune Response and Potential Implications for Treating Orthopedic Disorders.” Frontiers in Molecular Biosciences 9: 1065265. 36545506 10.3389/fmolb.2022.1065265 PMC9762617

[fsn371152-bib-0019] Liu, Z. , X. Ding , J. Wu , et al. 2019. “Dose‐Response Relationship Between Higher Serum Calcium Level and Higher Prevalence of Hyperuricemia: A Cross‐Sectional Study.” Medicine (Baltimore) 98: e15611. 31096467 10.1097/MD.0000000000015611 PMC6531036

[fsn371152-bib-0020] Lonsdale, K. 1968. “Human Stones.” Science 159: 1199–1207. 4886077 10.1126/science.159.3820.1199

[fsn371152-bib-0021] Lonsdale, K. , and P. Mason . 1966. “Uric Acid, Uric Acid Dihydrate, and Urates in Urinary Calculi, Ancient and Modern.” Science 152: 1511–1512. 5327201 10.1126/science.152.3728.1511

[fsn371152-bib-0022] Ma, Y. , C. Cheng , Z. Jian , et al. 2024. “Risk Factors for Nephrolithiasis Formation: An Umbrella Review.” International Journal of Surgery 110: 5733–5744. 38814276 10.1097/JS9.0000000000001719 PMC11392093

[fsn371152-bib-0023] Marreiro, D. D. , K. J. Cruz , J. B. Morais , J. B. Beserra , J. S. Severo , and A. R. de Oliveira . 2017. “Zinc and Oxidative Stress: Current Mechanisms.” Antioxidants (Basel) 6: 24. 28353636 10.3390/antiox6020024 PMC5488004

[fsn371152-bib-0024] McCormick, N. , and H. K. Choi . 2024. “Recurrent Gout and Serum Urate‐Reply.” JAMA 331: 1768–1769. 38696204 10.1001/jama.2024.5719 PMC11884748

[fsn371152-bib-0025] Pieczynska, J. , S. Placzkowska , R. Sozanski , et al. 2019. “Is Maternal Dietary Selenium Intake Related to Antioxidant Status and the Occurrence of Pregnancy Complications?” Journal of Trace Elements in Medicine and Biology 54: 110–117. 31109600 10.1016/j.jtemb.2019.04.010

[fsn371152-bib-0026] Powell, S. R. 2000. “The Antioxidant Properties of Zinc.” Journal of Nutrition 130: 1447S–1454S. 10801958 10.1093/jn/130.5.1447S

[fsn371152-bib-0027] Rai, S. K. , T. T. Fung , N. Lu , et al. 2017. “The Dietary Approaches to Stop Hypertension (DASH) Diet, Western Diet, and Risk of Gout in Men: Prospective Cohort Study.” BMJ (Clinical Research Ed.) 357: j1794. 10.1136/bmj.j1794 PMC5423545 28487277

[fsn371152-bib-0028] Umeki, S. , R. Ohga , Y. Konishi , T. Yasuda , K. Morimoto , and A. Terao . 1986. “Oral Zinc Therapy Normalizes Serum Uric Acid Level in Wilson's Disease Patients.” American Journal of the Medical Sciences 292: 289–292. 3777013 10.1097/00000441-198611000-00007

[fsn371152-bib-0029] Vijayaraghavan, K. , S. Iyyam Pillai , and S. P. Subramanian . 2012. “Design, Synthesis and Characterization of Zinc‐3 Hydroxy Flavone, a Novel Zinc Metallo Complex for the Treatment of Experimental Diabetes in Rats.” European Journal of Pharmacology 680: 122–129. 22327044 10.1016/j.ejphar.2012.01.022

[fsn371152-bib-0030] Yokose, C. , N. McCormick , N. Lu , et al. 2023. “Trends in Prevalence of Gout Among US Asian Adults, 2011‐2018.” JAMA Network Open 6: e239501. 37083663 10.1001/jamanetworkopen.2023.9501 PMC10122173

[fsn371152-bib-0031] Yuan, S. , L. Yu , W. Gou , et al. 2022. “Health Effects of High Serum Calcium Levels: Updated Phenome‐Wide Mendelian Randomisation Investigation and Review of Mendelian Randomisation Studies.” eBioMedicine 76: 103865. 35134646 10.1016/j.ebiom.2022.103865 PMC8844774

[fsn371152-bib-0032] Zgaga, L. , E. Theodoratou , J. Kyle , et al. 2012. “The Association of Dietary Intake of Purine‐Rich Vegetables, Sugar‐Sweetened Beverages and Dairy With Plasma Urate, in a Cross‐Sectional Study.” PLoS One 7: e38123. 22701608 10.1371/journal.pone.0038123 PMC3368949

[fsn371152-bib-0033] Zhang, Y. , S. Chen , M. Yuan , Y. Xu , and H. Xu . 2022. “Gout and Diet: A Comprehensive Review of Mechanisms and Management.” Nutrients 14: 3525. 36079783 10.3390/nu14173525 PMC9459802

[fsn371152-bib-0034] Zhang, Y. , Y. Liu , and H. Qiu . 2018. “Association Between Dietary Zinc Intake and Hyperuricemia Among Adults in the United States.” Nutrients 10: 568. 29734733 10.3390/nu10050568 PMC5986448

[fsn371152-bib-0035] Zhang, Y. , and H. Qiu . 2018. “Dietary Magnesium Intake and Hyperuricemia Among US Adults.” Nutrients 10: 296. 29498657 10.3390/nu10030296 PMC5872714

