# Supplementary material for: The Association of Mixed Mineral Intake Exposure With Gout: A National Cross‐Sectional National Health and Nutrition Examination Survey (NHANES) Study
Source: Food Sci Nutr. 2025 Nov 9;13(11):e71152. doi: 10.1002/fsn3.71152 (PMC12597780; doi:10.1002/fsn3.71152)
Supplement: Supplementary file 1 — Table S1:Baseline characteristics of the study sample. Table S2: The weighted basic characteristics of the study sample after PSM. Table S3: Posterior inclusion probabilities (PIPs) estimates for all mineral intake exposures. Figure S1: Plot of standardized mean differences before and after propensity score matching for baseline covariates. Figure S2: Restricted cubic spline (RCS) plots of multimineral intake on gout. [file FSN3-13-e71152-s001.docx]

**Supplementary materials**

**Supplementary Table S1. Baseline characteristics of the study sample.**

| Variable | Total  22661 ( 100 % )1 | non-gout  21525 ( 94.99 % )1 | gout  1136 ( 5.01 % )1 | p-value2 | |
| --- | --- | --- | --- | --- | --- |
| age |  |  |  | <0.001 | |
| 16-59 | 15,293 (67.49%) | 14,907 (69.25%) | 386 (33.98%) |  | |
| 60+ | 7,368 (32.51%) | 6,618 (30.75%) | 750 (66.02%) |  | |
| Gender |  |  |  | <0.001 | |
| male | 11,908 (52.55%) | 11,054 (51.35%) | 854 (75.18%) |  | |
| female | 10,753 (47.45%) | 10,471 (48.65%) | 282 (24.82%) |  | |
| Race |  |  |  | <0.001 | |
| MexiCAn AmeriCAn | 3,170 (13.99%) | 3,096 (14.38%) | 74 (6.51%) |  | |
| Non-Hispanic White | 10,351 (45.68%) | 9,770 (45.39%) | 581 (51.14%) |  | |
| Non-Hispanic Black | 4,728 (20.86%) | 4,424 (20.55%) | 304 (26.76%) |  | |
| Other Race | 4,412 (19.47%) | 4,235 (19.67%) | 177 (15.58%) |  | |
| mar |  |  |  | <0.001 | |
| Married/Living with partner | 9,151 (40.38%) | 8,764 (40.72%) | 387 (34.07%) |  | |
| Widowed/Divorced/Separated/Never married | 13,510 (59.62%) | 12,761 (59.28%) | 749 (65.93%) |  | |
| edu |  |  |  | 0.2 | |
| <High school | 1,765 (7.79%) | 1,661 (7.72%) | 104 (9.15%) |  | |
| Completed high school | 3,092 (13.64%) | 2,938 (13.65%) | 154 (13.56%) |  | |
| >High school | 17,804 (78.57%) | 16,926 (78.63%) | 878 (77.29%) |  | |
| pir |  |  |  | 0.9 | |
| Low income | 6,831 (30.14%) | 6,495 (30.17%) | 336 (29.58%) |  | |
| middle income | 8,571 (37.82%) | 8,135 (37.79%) | 436 (38.38%) |  | |
| high income | 7,259 (32.03%) | 6,895 (32.03%) | 364 (32.04%) |  | |
| bmi |  |  |  | <0.001 | |
| normal | 6,365 (28.09%) | 6,195 (28.78%) | 170 (14.96%) |  | |
| heavy | 7,441 (32.84%) | 7,098 (32.98%) | 343 (30.19%) |  | |
| overweight | 8,855 (39.08%) | 8,232 (38.24%) | 623 (54.84%) |  | |
| diabetes |  |  |  | <0.001 | |
| diabetes no | 19,590 (86.45%) | 18,837 (87.51%) | 753 (66.29%) |  | |
| diabetes yes | 3,071 (13.55%) | 2,688 (12.49%) | 383 (33.71%) |  | |
| hbp |  |  |  | <0.001 | |
| hbp no | 12,293 (54.25%) | 12,085 (56.14%) | 208 (18.31%) |  | |
| hbp yes | 10,368 (45.75%) | 9,440 (43.86%) | 928 (81.69%) |  | |
| smoke |  |  |  | <0.001 | |
| never | 11,319 (49.95%) | 10,894 (50.61%) | 425 (37.41%) |  | |
| former | 6,169 (27.22%) | 5,668 (26.33%) | 501 (44.10%) |  | |
| now | 5,173 (22.83%) | 4,963 (23.06%) | 210 (18.49%) |  | |
| Alcohol |  |  |  | <0.001 | |
| drinkers | 18,761 (82.79%) | 17,970 (83.48%) | 791 (69.63%) |  | |
| non-drinkers | 3,900 (17.21%) | 3,555 (16.52%) | 345 (30.37%) |  | |
| Calcium(mg) | 816 (531, 1,190) | 821 (534, 1,198) | 732 (469, 1,046) | <0.001 | |
| Phosphorus(mg) | 1,250 (903, 1,702) | 1,253 (906, 1,706) | 1,205 (855, 1,625) | | <0.001 |
| Magnesium(mg) | 271 (196, 370) | 272 (197, 371) | 265 (187, 355) | 0.004 | |
| Iron(mg) | 13 (9, 18) | 13 (9, 18) | 12 (9, 17) | 0.064 | |
| Zinc(mg) | 10 (7, 14) | 10 (7, 14) | 9 (6, 14) | 0.012 | |
| Copper(mg) | 1.10 (0.79, 1.53) | 1.10 (0.79, 1.53) | 1.09 (0.77, 1.49) | 0.2 | |
| Sodium(mg) | 3,203 (2,274, 4,395) | 3,206 (2,278, 4,403) | 3,141 (2,226, 4,256) | 0.049 | |
| Potassium(mg) | 2,459 (1,766, 3,301) | 2,458 (1,768, 3,301) | 2,479 (1,740, 3,292) | 0.5 | |
| Selenium(mcg) | 103 (71, 144) | 103 (71, 144) | 102 (70, 142) | 0.4 | |
| ^1^n (%); Median (Q1, Q3) | | | | | |
| ^2^Pearson's Chi-squared test; Wilcoxon rank sum test | | | | | |

*edu, education; pir, poverty income ratio; bmi, body mass index; hbp, high blood pressure.

**Supplementary Table S2. The weighted basic characteristics of the study sample after PSM.**

| **Variable** | **Total  2272 ( 100 % )^1^** | **non-gout  1136 ( 50 % )^1^** | **gout  1136 ( 50 % )^1^** | **p-value^2^** |
| --- | --- | --- | --- | --- |
| age |  |  |  | 0.8 |
| 16-59 | 765 (33.67%) | 379 (33.36%) | 386 (33.98%) |  |
| 60+ | 1,507 (66.33%) | 757 (66.64%) | 750 (66.02%) |  |
| Gender |  |  |  | 0.5 |
| female | 549 (24.16%) | 267 (23.50%) | 282 (24.82%) |  |
| male | 1,723 (75.84%) | 869 (76.50%) | 854 (75.18%) |  |
| Race |  |  |  | 0.018 |
| Mexican American | 186 (8.19%) | 112 (9.86%) | 74 (6.51%) |  |
| Non-Hispanic Black | 589 (25.92%) | 285 (25.09%) | 304 (26.76%) |  |
| Non-Hispanic White | 1,128 (49.65%) | 547 (48.15%) | 581 (51.14%) |  |
| Other Race | 369 (16.24%) | 192 (16.90%) | 177 (15.58%) |  |
| mar |  |  |  | 0.4 |
| Married/Living with partner | 757 (33.32%) | 370 (32.57%) | 387 (34.07%) |  |
| Widowed/Divorced/Separated/Never married | 1,515 (66.68%) | 766 (67.43%) | 749 (65.93%) |  |
| edu |  |  |  | 0.7 |
| <High school | 198 (8.71%) | 94 (8.27%) | 104 (9.15%) |  |
| >High school | 1,762 (77.55%) | 884 (77.82%) | 878 (77.29%) |  |
| Completed high school | 312 (13.73%) | 158 (13.91%) | 154 (13.56%) |  |
| pir |  |  |  | 0.3 |
| high income | 737 (32.44%) | 373 (32.83%) | 364 (32.04%) |  |
| Low income | 640 (28.17%) | 304 (26.76%) | 336 (29.58%) |  |
| middle income | 895 (39.39%) | 459 (40.40%) | 436 (38.38%) |  |
| bmi |  |  |  | 0.085 |
| heavy | 728 (32.04%) | 385 (33.89%) | 343 (30.19%) |  |
| normal | 313 (13.78%) | 143 (12.59%) | 170 (14.96%) |  |
| overweight | 1,231 (54.18%) | 608 (53.52%) | 623 (54.84%) |  |
| diabetes |  |  |  | 0.079 |
| diabetes no | 1,545 (68.00%) | 792 (69.72%) | 753 (66.29%) |  |
| diabetes yes | 727 (32.00%) | 344 (30.28%) | 383 (33.71%) |  |
| hbp |  |  |  | 0.9 |
| hbp no | 419 (18.44%) | 211 (18.57%) | 208 (18.31%) |  |
| hbp yes | 1,853 (81.56%) | 925 (81.43%) | 928 (81.69%) |  |
| smoke |  |  |  | 0.9 |
| former | 995 (43.79%) | 494 (43.49%) | 501 (44.10%) |  |
| never | 848 (37.32%) | 423 (37.24%) | 425 (37.41%) |  |
| now | 429 (18.88%) | 219 (19.28%) | 210 (18.49%) |  |
| Alcohol |  |  |  | 0.8 |
| drinkers | 1,587 (69.85%) | 796 (70.07%) | 791 (69.63%) |  |
| non-drinkers | 685 (30.15%) | 340 (29.93%) | 345 (30.37%) |  |
| Calcium(mg) | 767 (501, 1,120) | 810 (520, 1,171) | 732 (469, 1,046) | <0.001 |
| Phosphorus(mg) | 1,228 (885, 1,659) | 1,266 (900, 1,696) | 1,205 (855, 1,625) | 0.009 |
| Magnesium(mg) | 268 (195, 363) | 275 (200, 372) | 265 (187, 355) | 0.004 |
| Iron(mg) | 13 (9, 18) | 14 (9, 19) | 12 (9, 17) | 0.002 |
| Zinc(mg) | 9.7 (6.6, 14.0) | 10.0 (6.7, 14.4) | 9.4 (6.5, 13.6) | 0.022 |
| Copper(mg) | 1.11 (0.77, 1.51) | 1.12 (0.80, 1.53) | 1.09 (0.77, 1.49) | 0.12 |
| Sodium(mg) | 3,172 (2,267, 4,325) | 3,222 (2,317, 4,407) | 3,141 (2,226, 4,256) | 0.072 |
| Potassium(mg) | 2,459 (1,766, 3,301) | 2,458 (1,768, 3,301) | 2,479 (1,740, 3,292) | 0.5 |
| Selenium(mcg) | 103 (72, 142) | 105 (73, 143) | 102 (70, 142) | 0.4 |
| ^1^n (%); | | | | |
| ^2^Pearson's Chi-squared test; Wilcoxon rank sum test | | | | |

*edu, education; pir, poverty income ratio; bmi, body mass index; hbp, high blood pressure.

**Supplementary Figure S1. Plot of Standardized Mean Differences Before and After Propensity Score Matching for Baseline Covariates.
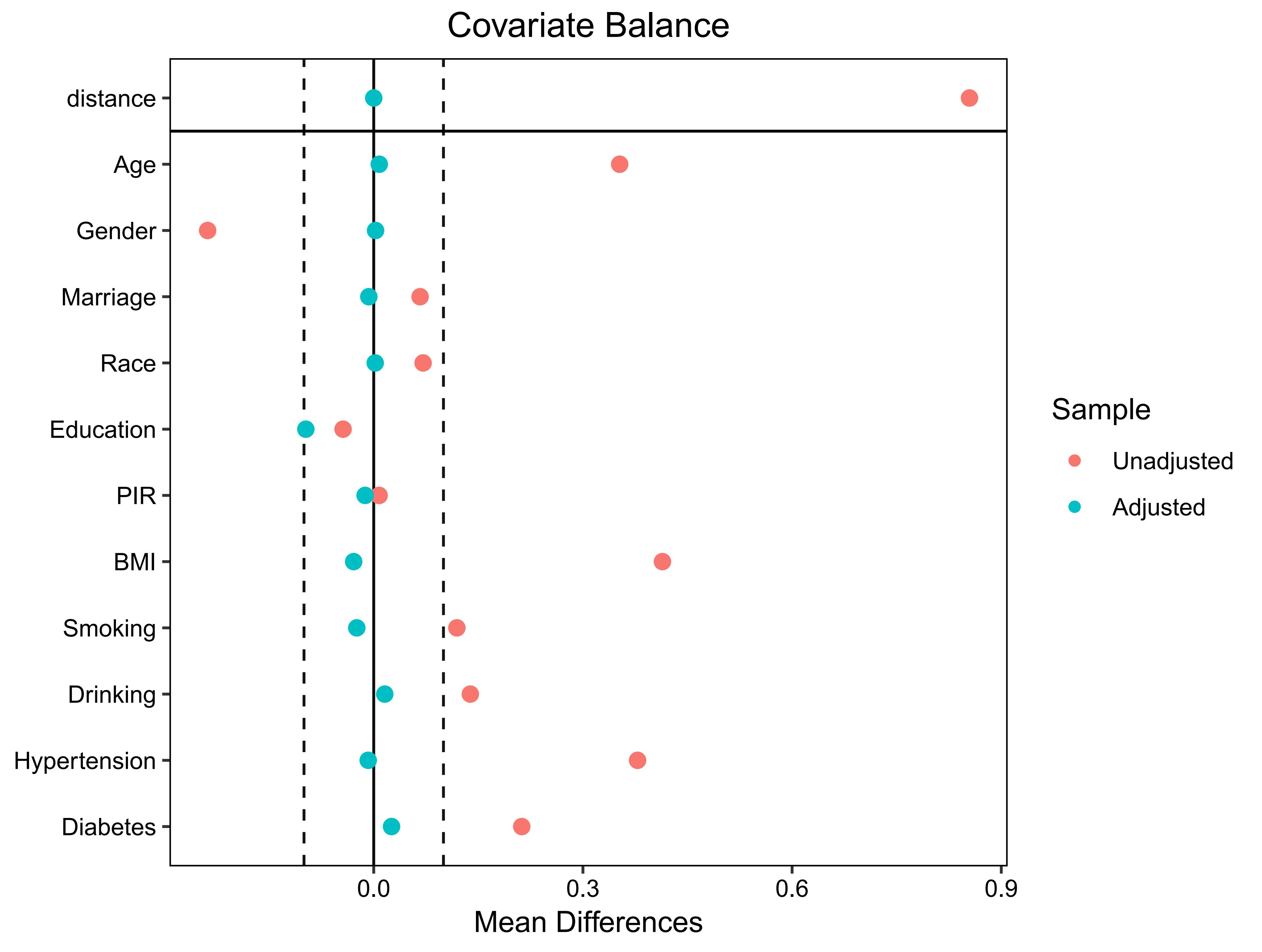
**

*Standardized Mean Differences Before and After Propensity Score Matching for Baseline Covariates. Red and blue dots represent unadjusted and adjusted mean differences, respectively; vertical dashed lines denote the ±0.1 balance threshold.

**Supplementary Table S3. Posterior inclusion probabilities(PIPs) Estimates for All Mineral Intake Exposures.**

| **Exposure** | Calcium | Phosphorus | Magnesium | Iron | Zinc | Copper | Sodium | Potassium | Selenium |
| --- | --- | --- | --- | --- | --- | --- | --- | --- | --- |
| **PIP** | 0.722 | 0.2184 | 0.2188 | 0.3974 | 0.211 | 0.1878 | 0.198 | 0.2586 | 0.176 |

**Supplementary Figure S2. Restricted cubic spline(RCS) plots of multi-mineral intake on gout.**
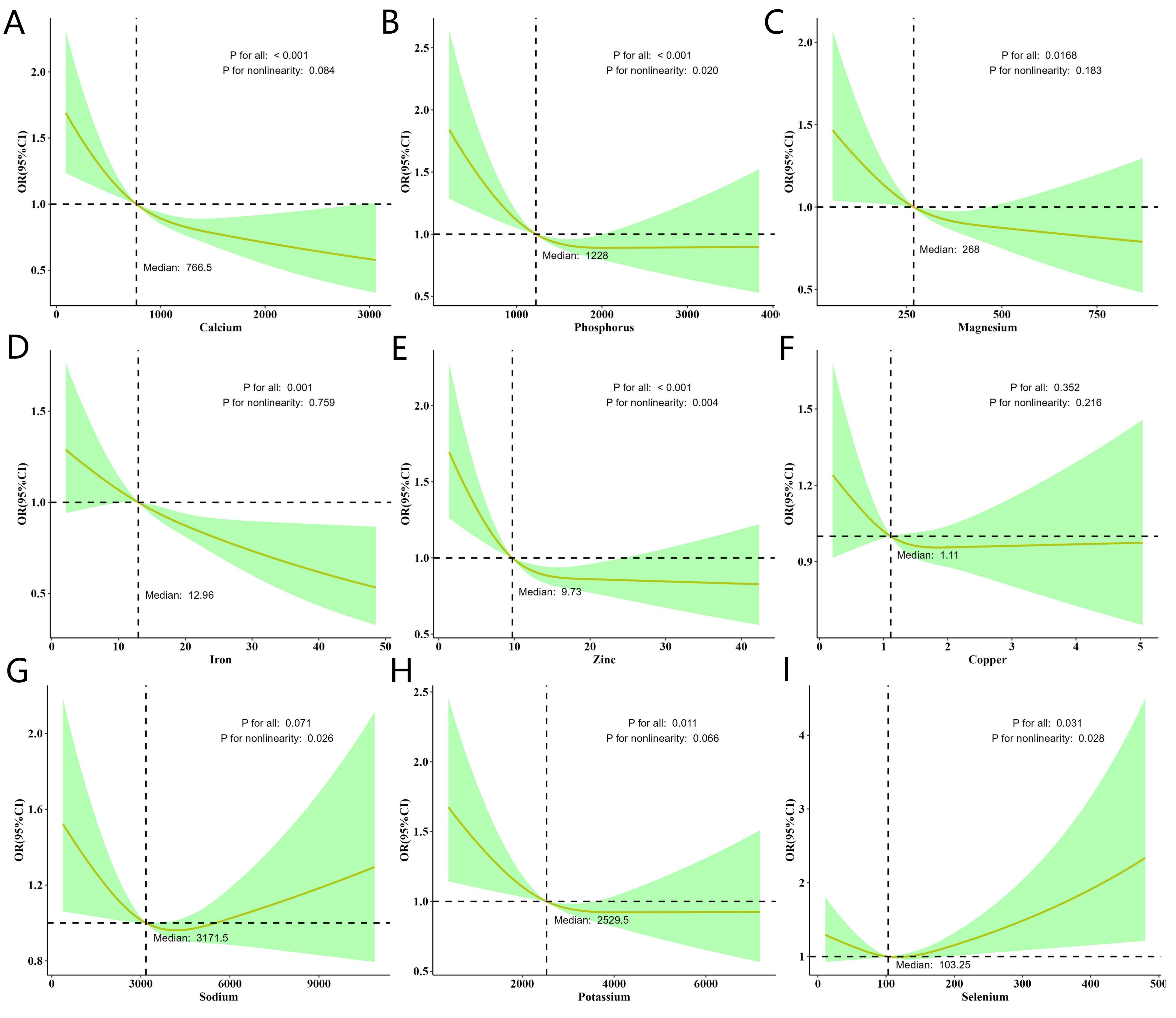


*(A) Calcium intake on Gout. (B) Phosphorus intake on Gout. (C) Magnesium intake on Gout. (D) Iron intake on Gout. (E) Zinc intake on Gout. (F) Potassium intake on Gout. (G) Sodium intake on Gout. (H) Copper intake on Gout. (I) Selenium intake on Gout.
